# Supplementary material for: Crystal structures of fukutin-related protein (FKRP), a ribitol-phosphate transferase related to muscular dystrophy
Source: Nat Commun. 2020 Jan 16;11:303. doi: 10.1038/s41467-019-14220-z (PMC6965139; doi:10.1038/s41467-019-14220-z)
Supplement: Supplementary file 1 — Supplementary Information [file 41467_2019_14220_MOESM1_ESM.pdf]

Supplementary Information

Crystal structures of fukutin-related protein (FKRP), a ribitol-phosphate transferase related to muscular dystrophy

Naoyuki Kuwabara, Rieko Imae *et al.*

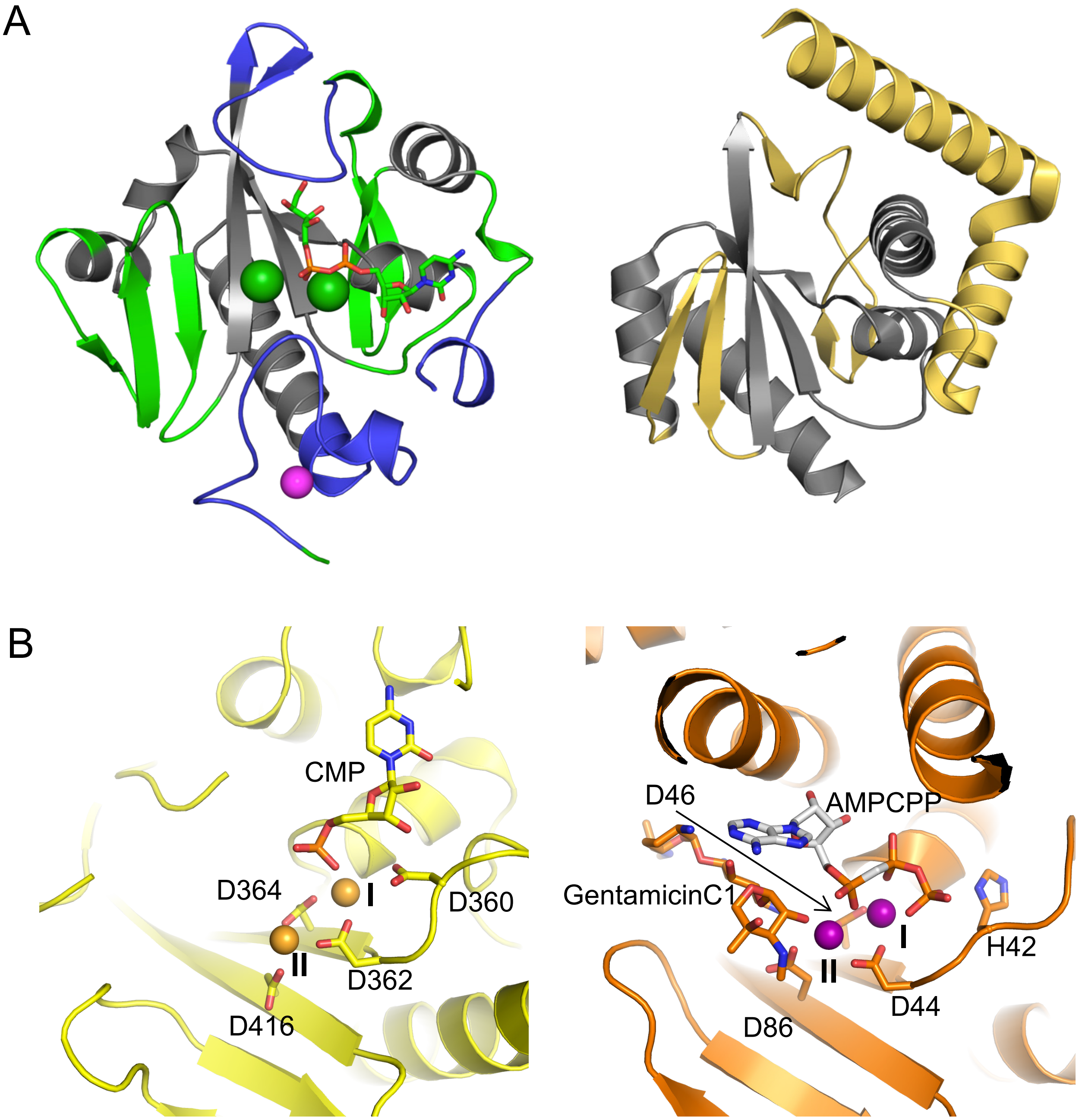

Supplementary Figure 1.
Structural comparison of FKRP and NTase. A) Catalytic domains of FKRP (left panel) and ANT(2")-Ia (right panel, PDB ID; 5KQJ) as a representative NTase are shown. The bound Ba^{2+}, Zn^{2+} and CDP-Rbo are shown as green spheres, a purple sphere and a stick model, respectively. The FKRP-specific structures (the zinc finger loop, a loop ordered by acceptor binding, and the C-terminal loop region) are colored blue. In both panels, the NTase core domains are colored grey. B) Structural comparison around the active sites. The left panel shows the structure of active site of sFKRP in Mg^{2+} (orange balls) and CMP-bound state. D360, D362, D364, D416 and CMP are shown as stick models. The right panel shows the structure of the active site of ANT(2")-Ia in gentamicin C1, Mn^{2+} (purple balls) and AMPCPP (adenosine-5′-[(α,β)-methyleno]-triphosphate) bound state (PDB ID; 5CFT). Carbon atoms are shown in white in AMPCPP. H42, D44, D46, D86 and kanamycin are shown as stick models. The residues excluding H42 (which corresponds to D360 in FKRP, and does not interact with Mn^{2+}) are conserved.

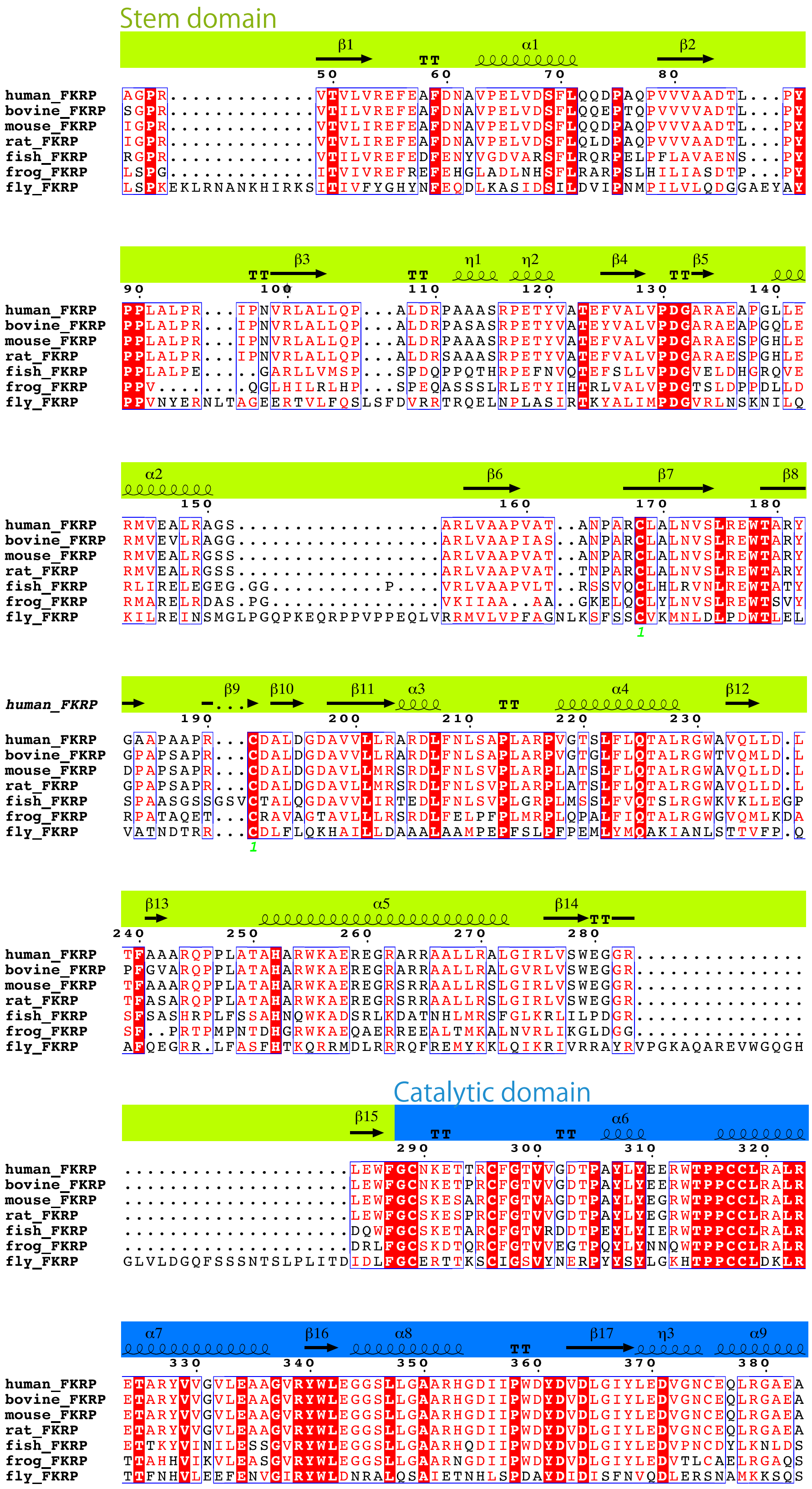

Supplementary Figure 2

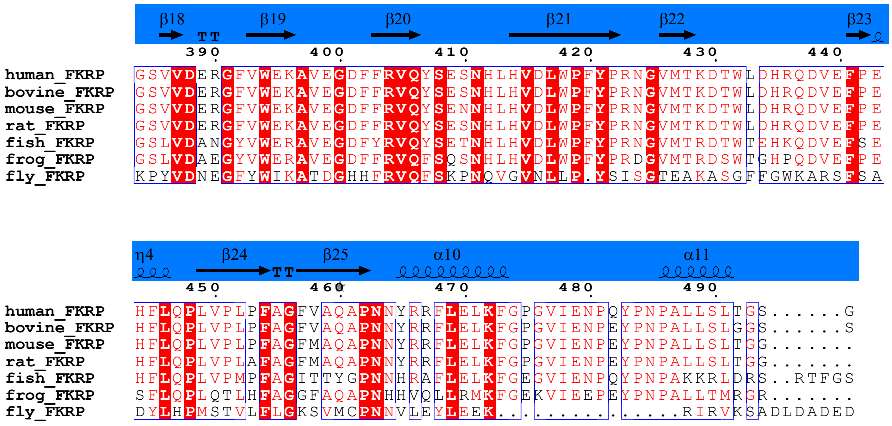

Supplementary Figure 2.
Structure-based sequence alignment of FKRP homologues, calculated using the program MAFFT [1]. White letters on red boxes represent identical amino-acid residues; red letters boxed by blue represent homologous residues. Arrows (β-strand), TTs (turn), coil symbols (α-helix) above the sequences, and green 1 characters (S-S bond) below the sequence are determined and drawn using ENDscript server [2] and the program ESPRIPT 3.0 (http://endscript.ibcp.fr). The aligned proteins sequences are from human (Q9H955), bovine (F1MN71), mouse (Q8CG64), rat (Q4KLJ4), fish (Q0PIP5), frog (A0A1L8F835), and fly (Q9W2P2). UniprotKB/Swiss-Prot entries are indicated in parentheses.

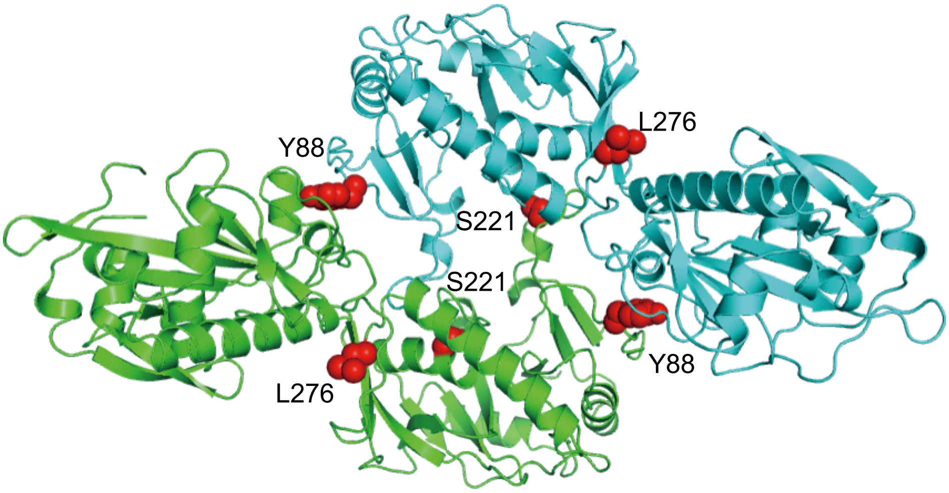

Supplementary Figure 3.
Mutation sites in the protomeric dimer. The dimer structure of sFKRP is shown in cartoon model. Three disease-related mutation sites analyzed in this study are shown in red spheres.

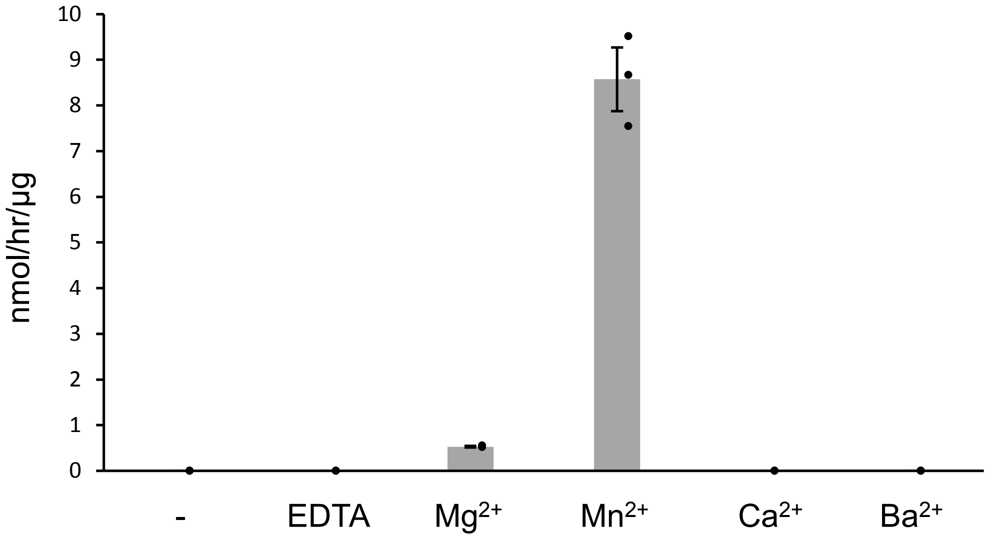

Supplementary Figure 4.
Divalent cation requirements for the enzymatic activity of sFKRP with CDP-Rbo and the RboP-(phospho-)core M3 peptide. A reaction buffer containing no divalent cations or containing 10 mM EDTA, $MgCl_2$, $MnCl_2$, $CaCl_2$ or $BaCl_2$ was used respectively. Average values ± SE of three independent experiments are shown. Each dot represents one data point. Source data are provided as a Source Data file.

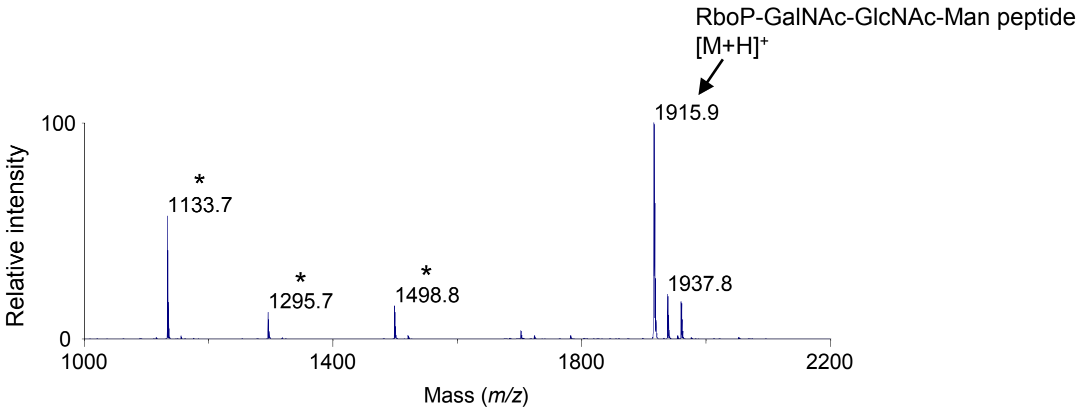

Supplementary Figure 5.
Confirmation of the preparation of the RboP-core M3 peptide by MALDI-TOF-MS. The peak 1915.9 corresponds to the $[M + H]^+$ ion of the RboP-core M3 peptide (RboP-GalNAc-GlcNAc-Man peptide). The peak 1937.8 corresponds to the $[M + Na]^+$ ion of the same peptide. Asterisks represent fragment ions formed during the MS experiment.

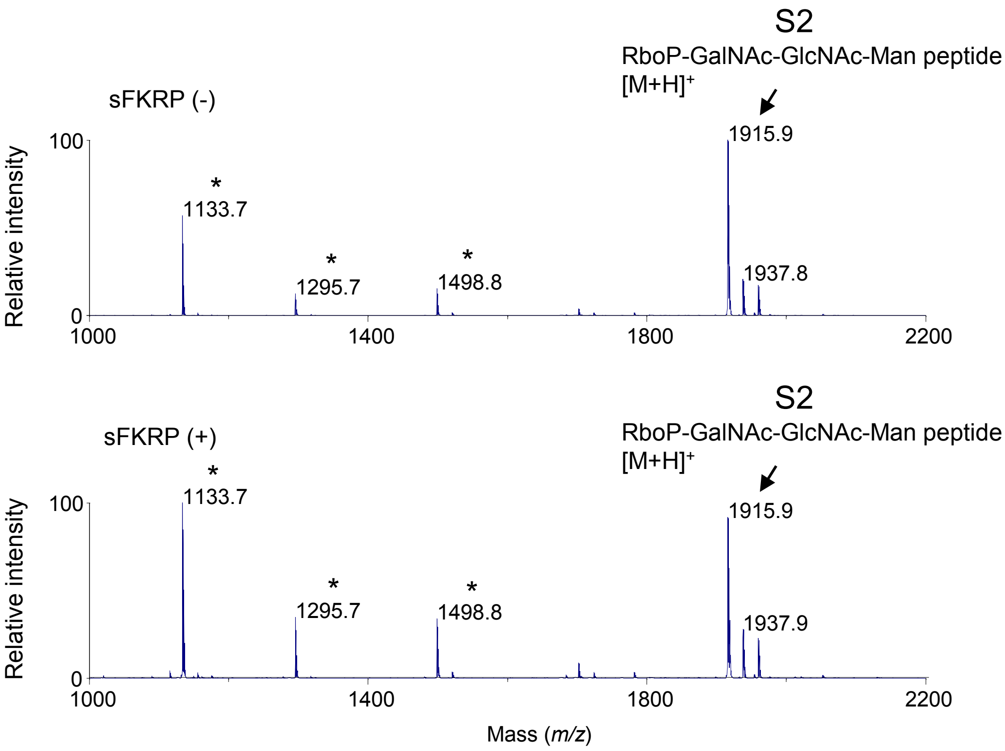

Supplementary Figure 6.

MALDI-TOF-MS spectra of the eluate around peak S2 in Figure 5B. The peak 1915.9 corresponding to the $[M + H]^+$ ion of the RboP-core M3 peptide (RboP-GalNAc-GlcNAc-Man peptide) was detected but the peak corresponding to the RboP-transferred RboP-RboP-core M3 peptide (calculated *m/z* value, 2129.9) was not detected in the presence of sFKRP (lower). Asterisks represent fragment ions formed during the MS experiment.

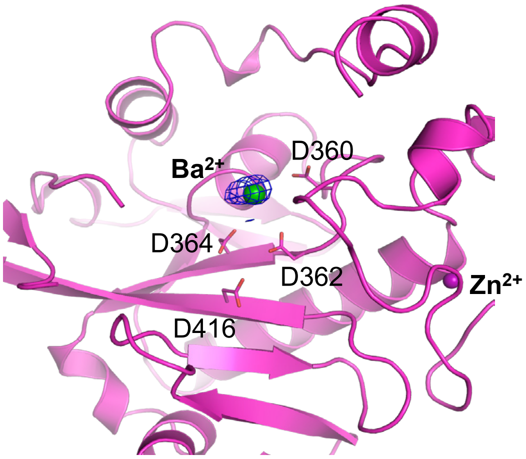

Supplementary Figure 7.
The anomalous difference Fourier map around the Barium ion for the Ba-SAD data set (blue mesh). The contour level is 5.0 σ. The color configuration is the same as in Figure 3B.

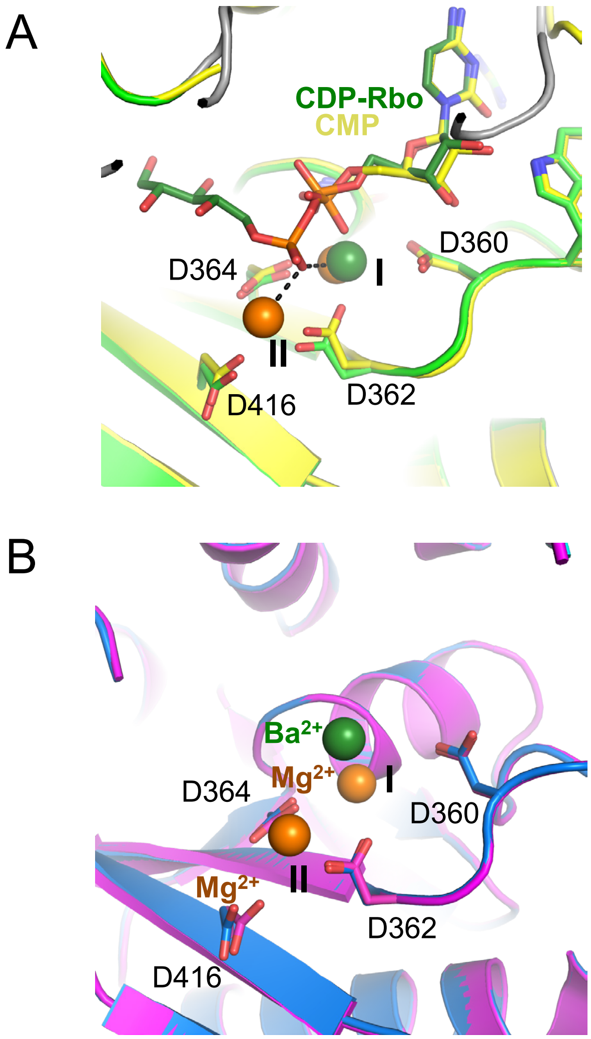

Supplementary Figure 8.
A) Superimposition of the CDP-Rbo-bound and CMP-bound structures. The color configuration is the same as in Figure 3C and D, except for CMP and CDP-Rbo. Carbon atoms in CDP-Rbo and CMP are colored green and yellow, respectively. Dotted lines indicate proximity between CDP-Rbo (in the Ba^{2+} and CDP-Rbo-bound structure) and Mg^{2+} (the Mg^{2+} and CMP-bound structure). The distances between Ob and Mg^{2+} (site I), and between Ob and Mg^{2+} (site II) were 2.9 and 2.8 Å, respectively. B) Comparison of substrate-free forms. The Mg^{2+}-bound structure (Figure 3A) and Ba^{2+}-bound structure (Figure 3B) are superimposed. At site I, the long coordination distances observed in the Mg^{2+}-bound form permit the binding of Ba^{2+}, which has a larger ionic radius. However, site II has a small coordination sphere and it cannot accommodate a Ba^{2+}.

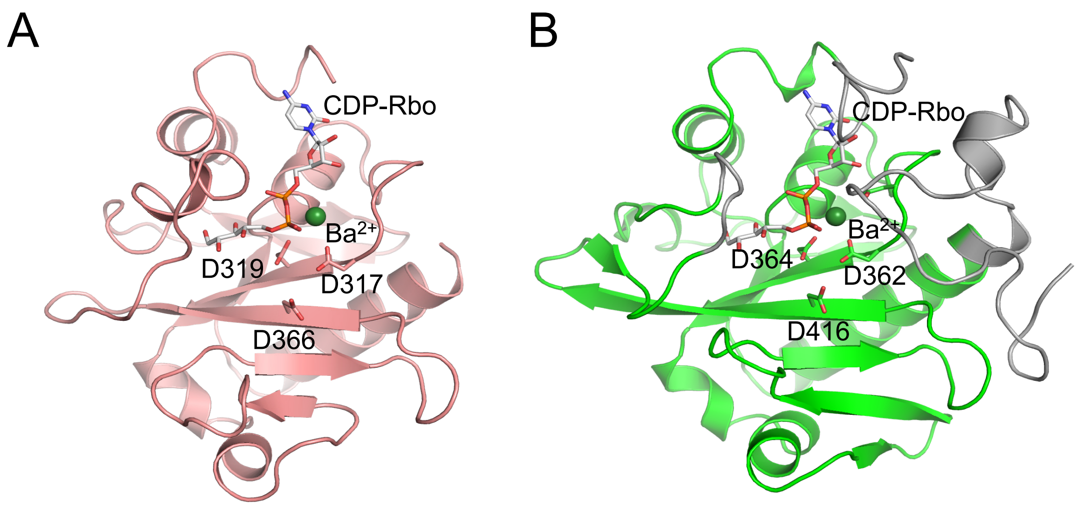

Supplementary Figure 9.
A) Homology model of the catalytic domain of FKTN (E249–Y461). Three Asp residues (D317, D319, and D366) are shown as stick models. CDP-Rbo and Ba^{2+} were placed according to the template structure. This model was prepared by SWISS-MODEL (https://swissmodel.expasy.org). B) Structure of the catalytic domain of FKRP (G288-L491) with CDP-Rbo and Ba^{2+} used as a template model. The colors are assigned as in Figure 3C.

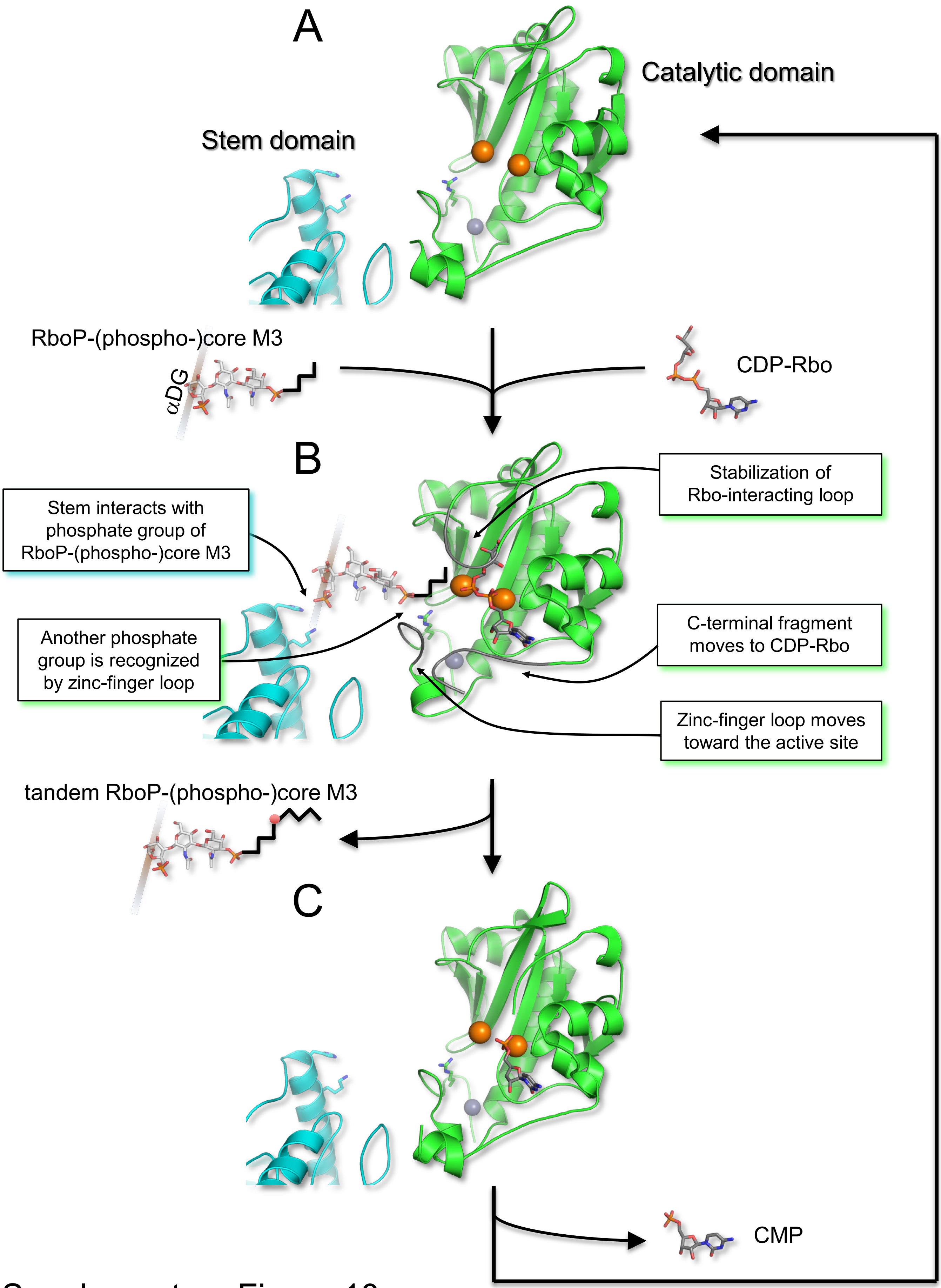

Supplementary Figure 10

Supplementary Figure 10.

Proposed catalytic mechanism of FKRP revealed in this study. In this figure, the catalytic domain (green) and stem domain from another protomer (light blue) are picked up for clarity. Mg^{2+} and Zn^{2+} are shown as orange and gray balls, respectively. A) In the substrate-free state (two Mg^{2+} are bound in this figure, versus one Ba^{2+} at site I), the Rbo-interacting loop is disordered and the C-terminal fragment locates far from the CDP-Rbo binding site. B) When CDP-Rbo binds, the Rbo-interacting loop covers the Rbo moiety and the C-terminal fragment moves to interact with the CDP moiety. In accompaniment with these conformational changes, the zinc-finger loop moves toward the active site to form an acceptor-binding site. The acceptor glycopeptide interacts with both the catalytic and stem domains from different protomers. Then, RboP is transferred to RboP-(phospho-)core M3 from CDP-Rbo. The regions where conformational changes are evoked are colored grey. R295 is shown by stick model. C) After releasing the product (tandem RboP-(phospho-)core M3), the conformation of FKRP returns to the substrate-free form to release CMP.

Supplementary Table 1. X-ray crystallographic data collection and refinement statistics

data statistics	Ba^{2+} bound form	Ba-SAD data	Mg^{2+} bound form	Mg^{2+} and CMP complex	Ba^{2+} and CDP-ribitol complex	Ba^{2+}, CDP-ribitol and acceptor complex
**Wavelength (angstrom)**	1.0000	1.9000	1.0000	1.0000	1.0000	0.9800
**Resolution range**	48.92 - 2.25 (2.33 - 2.25)	48.93 - 2.24 (2.28-2.24)	45.65 - 2.06(2.13 - 2.06)	47.13 - 2.60(2.69 - 2.60)	43.31 - 2.23 (2.31 - 2.23)	42.74 - 2.47 (2.55 - 2.47)
**Space group**	$P2_12_12_1$	$P2_12_12_1$	$P2_12_12_1$	$P2_12_12_1$	$P2_12_12_1$	$P2_12_12_1$
**Unit cell a,b,c**	77.1, 119.0, 257.6	77.2, 119.2, 255.8	76.8, 119.4,258.1	77.6, 118.7, 252.7	77.4, 118.8, 253.1	78.6, 119.2, 254.6
**Total reflections**	836111	3249448	1025492	485564	752011	574111
**Unique reflections**	112891	114256	147623	72606	114810	86984
**Multiplicity**	7.4	28.4	6.9	6.7	6.5	6.6
**Completeness**	1.00 (0.99)	1.00 (1.00)	1.00 (0.97)	1.00 (1.00)	1.00 (0.97)	1.00 (0.99)
**Mean I/sigma(I)**	14.47 (1.59)	22.40 (2.40)	18.68 (1.75)	13.56 (1.56)	14.36 (1.61)	16.18 (1.62)
**Wilson B-factor**	45.39	43.36	37.00	51.64	39.56	55.28
**R-merge**	0.099	0.140	0.066	0.119	0.098	0.087
**R-meas**	0.107	0.143	0.071	0.130	0.106	0.094
**CC1/2**	0.999 (0.708)	0.999 (0.838)	0.999 (0.633)	0.998 (0.718)	0.999 (0.699)	0.999 (0.765)
**refinement statistics**						
**PDB ID**	6KAN	6L7U	6KAK	6KAL	6KAJ	6KAM
**R-work**	0.196	0.200	0.196	0.195	0.209	0.208
**R-free**	0.232	0.234	0.227	0.241	0.244	0.249
**Number of non-hydrogen atoms**	14132	14108	14589	14174	14571	14279
**macromolecules**	13541	13531	13523	13805	13780	13796
**ligands**	107	78	123	110	256	290
**solvent**	484	499	943	259	535	193
**Protein residues**	1762	1758	1767	1772	1779	1779
**RMS(bonds)**	0.010	0.005	0.006	0.010	0.005	0.007
**RMS(angles)**	1.05	0.767	0.724	1.002	0.868	0.949
**Ramachandran favored (%)**	97.6	97.1	97.8	96.8	96.9	96.8
**Ramachandran allowed (%)**	2.3	2.7	2.1	3.0	3.0	3.1
**Ramachandran outliers (%)**	0.1	0.2	0.1	0.2	0.1	0.2
**Rotamer outliers (%)**	0.2	0.1	0.0	0.1	0.0	0.0
**Average B-factor**	57.47	59.56	46.61	65.16	56.71	75.35
**macromolecules**	57.52	59.67	46.44	65.27	56.82	75.09
**ligands**	82.05	84.35	65.02	80.53	68.38	97.92
**solvent**	50.39	52.70	46.69	52.99	49.38	60.36

Statistics for the highest-resolution shell are shown in parentheses.

Supplementary Table 1. X-ray crystallographic data collection and refinement statistics (continued)

data statistics	Mg^{2+} bound form<br>Zinc peak data*	Mg^{2+} bound form<br>Zinc remote low data*
Wavelength (angstrom)	1.2825	1.2898
Resolution range	47.30 - 2.41 (2.49 - 2.41)	49.05 - 2.41 (2.49 - 2.41)
Space group	$P2_12_12_1$	$P2_12_12_1$
Unit cell	76.9, 119.4, 257.6	77.0, 119.5, 257.8
Total reflections	610687	611519
Unique reflections	177224	177585
Multiplicity	3.4	3.4
Completeness (%)	1.00 (0.98)	1.00 (0.98)
Mean I/sigma(I)	12.97 (2.18)	12.15 (1.74)
Wilson B-factor	38.25	40.49
R-merge	0.074	0.081
R-meas	0.088	0.096
CC1/2	0.998 (0.788)	0.998 (0.694)
**refinement statistics**		
PDB ID	6L7S	6L7T
R-work	0.182	0.177
R-free	0.211	0.210
Number of non-hydrogen atoms	14524	14642
macromolecules	13562	13562
ligands	108	108
solvent	854	972
Protein residues	1759	1759
RMS(bonds)	0.007	0.006
RMS(angles)	0.782	0.832
Ramachandran favored (%)	97.4	97.7
Ramachandran allowed (%)	2.6	2.2
Ramachandran outliers (%)	0.1	0.1
Rotamer outliers (%)	0.0	0.2
Average B-factor	49.5	49.95
macromolecules	49.48	49.81
ligands	66.54	65.87
solvent	47.41	50.11

Statistics for the highest-resolution shell are shown in parentheses.

* Friedel mates were counted separately.

Supplementary Table 2. Buried Surface Area (Å^2)

chain	**A**	**B**	**C**	**D**	**A+B**	**C+D**
**A**	-	1496 (931)	774 (0)	85(9)	-	859 (9)
**B**	1496 (931)	-	91(24)	697 (697)	-	788(721)
**C**	774 (0)	91(24)	-	1463(926)	865 (24)	-
**D**	85(9)	697 (697)	1463(926)	-	782 (706)	-
**A+B**	-	-	865 (24)	782 (706)	-	1647 (730)
**C+D**	859 (9)	788(721)	-	-	1647 (730)	-

Supplementary Table 3. Summary of metal ion interaction in each state

	Ligand bound state			
	Mg^{2+} bound		Mg^{2+} and CMP	
Mg^{2+} (site I) *1	Asp360(Oδ)	3.61	Asp360(Oδ)	3.37
	Asp362(Oδ)	3.40	Asp362(Oδ)	2.16
	Asp364(Oδ)	3.00	Asp364(Oδ)	2.06
			CMP (O1α)	2.61
Mg^{2+} (site II)	Asp362(Oδ)	2.22	Asp362(Oδ)	2.15
			Asp364(Oδ)	2.80
			Asp416(Oδ)	2.25

	Ligand bound state			
	Ba^{2+} bound		Ba^{2+} and CDP-Rbo	
Ba^{2+} (site I)	Asp360(Oδ)	4.38	Asp360(Oδ)	3.05
	Asp362(Oδ)	3.92	Asp362(Oδ)	2.78
	Asp364(Oδ)	3.90	Asp364(Oδ)	2.90
			CDP-Rbo(Oα)	2.72
			CDP-Rbo(Oβ)	2.96
Ba^{2+} (site II) *2	Asp362(Oδ)	2.98		
	Asp364(Oδ)	3.18	Not found	
	Asp416(Oδ)	4.34		

*1) Mg^{2+} (site I) in Mg^{2+} bound state is only found in chain A and B.
*2) Ba^{2+} (site II) in Ba^{2+} bound state is only found in chain B.

Supplementary Table 4. Sequences of used primers

**Oligonucleotide**	**Sequence 5'-3'**
FKRP_pPA_A45_F	GTACTTCCAGGGAGGCCGGCCTGCCGGCCCCCGTGTCACCGTCC
FKRP_pPA_Ct(G495)_R	CTTAAGCGCTAGAGGCCGGCCTCAGCCGCTTCCCGTCAGACTCAG
Y88F-F	GTGGCAGCCGACACGCTCCCCttcCCGCCCCTGGCCCTGCCCCGC
Y88F-R	GCGGGGCAGGGCCAGGGGCGGgaaGGGGAGCGTGTCGGCTGCCAC
S221R-F	CGCGCCCGCGACCTCTTCAACCTCagaGCGCCCCTGGCCCGGCCGG
S221R-R	CCGGCCGGGCCAGGGGCGCtctGAGGTTGAAGAGGTCGCGGGCGCG
L276I-F	CTGCTCCGCGCGCTGGGCATCCGCattGTGAGCTGGGAAGGCGGGCGGCTG
L276I-R	CAGCCGCCCGCCTTCCCAGCTCACaatGCGGATGCCCAGCGCGCGGAGCAG
D360A-F	GGACATCATCCCATGGgccTACGACGTGGACCTGG
D360A-R	CCAGGTCCACGTCGTAggcCCATGGGATGATGTCC
D362A-F	CGGGGACATCATCCCATGGGACTACgccGTGGACCTGGGCATCTACTTGGAGG
D362A-R	CCTCCAAGTAGATGCCCAGGTCCACggcGTAGTCCCATGGGATGATGTCCCCG
D364A-F	CATCATCCCATGGGACTACGACGTGgccCTGGGCATCTACTTGGAGGACGTGG
D364A-R	CCACGTCCTCCAAGTAGATGCCCAGggcCACGTCGTAGTCCCATGGGATGATG
D416A-F	CAGCGAAAGCAACCACTTGCACGTGgccCTGTGGCCCTTCTACCCCCGCAATG
D416A-R	CATTGCGGGGGTAGAAGGGCCACAGggcCACGTGCAAGTGGTTGCTTTCGCTG
H252A-F	GCCCCCGCTGGCCACGGCCgcgGCGCGCTGGAAGGCTGAGCG
H252A-R	CGCTCAGCCTTCCAGCGCGCcgcGGCCGTGGCCAGCGGGGGC
K256A-F	CCACGGCCCACGCGCGCTGGgcgGCTGAGCGCGAGGGACGCGC
K256A-R	GCGCGTCCCTCGCGCTCAGCcgcCCAGCGCGCGTGGGCCGTGG
R295A-F	GCTGCAACAAGGAGACCACGgcgTGCTTCGGAACCGTGGTGGG
R295A-R	CCCACCACGGTTCCGAAGCAcgcCGTGGTCTCCTTGTTGCAGC

Mutational codons were indicated as lower cases.

Supplementary References

1. Katoh K, Rozewicki J, Yamada KD (2017) MAFFT online service: multiple sequence alignment, interactive sequence choice and visualization. *Briefings in Bioinformatics*, 1ï 7. [doi: 10.1093/bib/bbx108]

2. Robert X, Gouet P (2014) Deciphering key features in protein structures with the new ENDscript server. *Nucleic Acids Res* 42:W320ï W324. [doi: 10.1093/nar/gku316]
